# A Meta-Analysis of *PTGS1* and *PTGS2* Polymorphisms and NSAID Intake on the Risk of Developing Cancer

**DOI:** 10.1371/journal.pone.0071126

**Published:** 2013-08-13

**Authors:** Mai Nagao, Youichi Sato, Aiko Yamauchi

**Affiliations:** Department of Pharmaceutical Information Science, Institute of Health Biosciences, The University of Tokushima Graduate School, Tokushima, Japan; MOE Key Laboratory of Environment and Health, School of Public Health, Tongji Medical College, Huazhong University of Science and Technology, China

## Abstract

**Background:**

Several studies have investigated whether the polymorphisms in the prostaglandin endoperoxide synthase 1 (*PTGS1*) and *PTGS2* genes and nonsteroidal anti-inflammatory drug (NSAID) use are associated with cancer risk; however, those studies have produced mixed results. Therefore, we performed a meta-analysis to evaluate the association between the *PTGS1* and *PTGS2* polymorphisms and the effect of NSAID use on the risk of developing cancer.

**Methods:**

We conducted a comprehensive search in PubMed through March 2012. The odds ratios (ORs) with the corresponding 95% confidence intervals (CIs) were calculated using the fixed-effect model or the random-effect model.

**Results:**

The database search generated 13 studies that met the inclusion criteria. For *PTGS1* rs3842787, NSAID users homozygous for the major allele (CC) had a significantly decreased cancer risk compared with non-NSAID users (OR = 0.73, 95% CI = 0.59–0.89). For *PTGS2* rs5275 and rs20417, there were no significant differences between the gene polymorphism and NSAID use on cancer risk among the 8 and 7 studies, respectively. However, in the stratified analysis by the type of cancer or ethnicity population, NSAID users homozygous for the major allele (TT) in rs5275 demonstrated significantly decreased cancer risk compared with non-NSAID users in cancer type not involving colorectal adenoma (OR = 0.70, 95% CI = 0.59–0.83) and among the USA population (OR = 0.67, 95% CI = 0.56–0.82). NSAID users homozygous for the major allele (GG) in rs20417 displayed a significantly decreased cancer risk than non-NSAID users among the US population (OR = 0.72, 95% CI = 0.58–0.88). For the *PTGS2* rs689466 and rs2745557 SNPs, there were no significant differences.

**Conclusion:**

This meta-analysis suggests that the associations between *PTGS* polymorphisms and NSAID use on cancer risk may differ with regard to the type of cancer and nationality.

## Introduction

Prostaglandin endoperoxide synthase 1 (*PTGS1*) and *PTGS2*, known as cyclooxygenase 1 (*COX1*) and *COX2*, catalyze the oxidative conversion of arachidonic acid to prostaglandin (PG) H_2_, which is subsequently metabolized to various biologically active metabolites, such as prostacyclin and thromboxane A_2_ [1]. Although both *PTGS1* and *PTGS2* catalyze the same committed step in prostanoid biosynthesis with similar efficiencies, they are encoded by distinct genes located on different chromosomes, and they substantially differ in their expression pattern [1]. *PTGS1* is constitutively expressed in most tissues and is responsible for the biosynthesis of PGs involved in various housekeeping functions, such as the regulation of renal, gastrointestinal, and platelet function [1]. *PTGS2* is rapidly induced by growth factors, inflammatory cytokines, and tumor promoters [2], and it primarily catalyzes PG synthesis in cells involved in both local and systemic inflammatory responses [1].

Inflammation increases the risk of several types of cancer, including colon, prostate, and pancreatic cancer [2], [3]. Therefore, it is postulated that reducing inflammation might decrease the development of cancer. Nonsteroidal anti-inflammatory drugs (NSAIDs) inhibit *PTGS*-mediated PG synthesis and reduce inflammation. NSAIDs are popular medicines used worldwide for the prevention and/or treatment of various diseases. Several epidemiological studies have investigated whether NSAID use is correlated to a reduced risk of developing cancer; however, this is a debatable matter. Furthermore, it is suggested that genetic variation in *PTGS1* and *PTGS2* might be related to cancer risk and/or drug efficacy in humans. To date, several studies have investigated associations of the polymorphisms in the *PTGS1* and *PTGS2* genes and NSAID use on cancer risk; however, these studies have produced mixed results. Therefore, we performed a meta-analysis to determine the association between the polymorphisms in *PTGS1* and *PTGS2* and NSAID use on the risk of developing cancer.

## Materials and Methods

### Literature Search

We searched for publications in MEDLINE, EMBASE, Science Direct and the Cochrane Library by using the keywords and strategy terms “cyclooxygenase” or “*COX*” or “*PTGS*”, “NSAID”, “genotype” or “polymorphism”, and “cancer” or “carcinoma” (last search was in March 2012). Non-controlled trials were excluded. Randomized controlled trials with three or more groups were retained if at least two groups addressed an eligible comparison.

### Inclusion Criteria

Studies were chosen if the following criteria were provided: (1) full-text articles were written in English; (2) controlled trials comparing *PTGS* polymorphisms and the risk of developing cancer, including NSAID use status; (3) sufficient published data for estimating an odds ratio (OR) or relative risk with 95% confidence interval (CI); and (4) the numbers of case, control, NSAID users, and non-NSAID-users by *PTGS* genotypes were clarified. The following information was not considered as selective criteria: (1) blindness of the trial; (2) type of cancer; (3) type of NSAID; and (4) NSAID dose method.

### Data Extraction

Data extraction was performed independently by two authors (Nagao and Sato) by using a standard protocol according to the criteria. The following data were extracted: the name of the first author, year of publication, country of research institution, type of cancer, study design, age, gender, and the number of cases and controls with NSAID users or non-users by genotype.

### Statistical Analysis

All statistical analyses were performed using the rmeta package for R, version 2.14.2 (The R Foundation for Statistical Computing, Tsukuba, Japan; http://www.R-project.org). Two-sided probability (*P*) values of <0.05 were considered statistically significant. ORs with 95% CIs were calculated to assess the strength of the following associations: (1) between *PTGS* genotype with NSAID users and the risk of developing cancer, (2) between NSAID users homozygous for the major allele and the risk of developing cancer, (3) between *PTGS* genotype with non-NSAID users and the risk of developing cancer, and (4) between NSAID users with minor allele carriers and the risk of developing cancer.

All meta-analyses were appraised for inter-study heterogeneity by using χ^2^-based Q statistics for statistical significance of heterogeneity. If there was no heterogeneity based on a Q-test *P* value more than 0.05, a fixed-effect model using the Mantel-Haenszel (M-H) method was used. Otherwise, the random-effects model using the DerSimonian and Laird method was employed. Sensitivity analyses were performed to assess the stability of the results by sequential omission of individual studies. To evaluate the possible publication bias, Egger’s test (linear regression method) and Begg’s test (rank correlation method) were used, and *P* values of <0.05 were considered representative of significant statistical publication bias.

## Results

### Characteristics of the Studies in Our Meta-analysis

**Figure 1:**
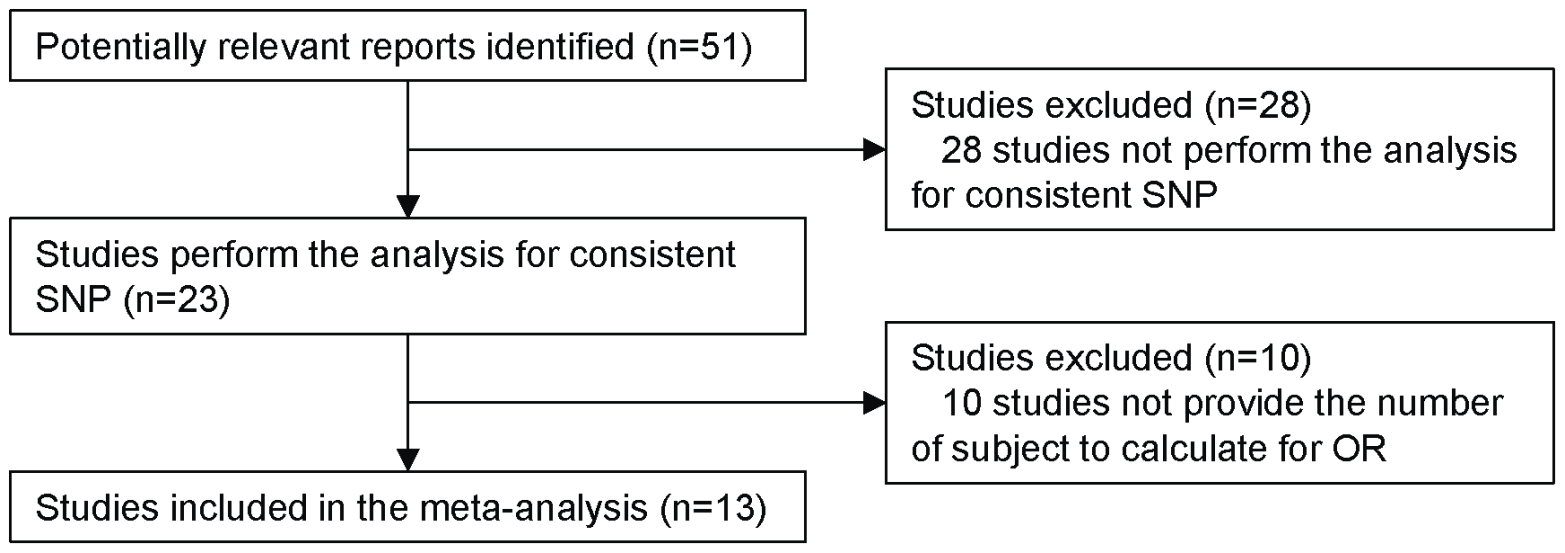
The flow diagram of the literature search and the study selection.

A total of 51 relevant reports were initially identified. Thirty-eight of the 51 studies were excluded because they did not meet our criteria. Among the 38 excluded studies, 28 studies did not perform the analysis for recurring SNPs, and 10 studies did not provide the number of subjects to calculate for OR. Therefore, 13 of the 51 studies were included in the meta-analysis (Fig. 1). All of the studies were published in English. The characteristics of the selected studies are summarized in Table 1 and Table S1. The 13 studies analyzed the following polymorphism: *PTGS1* rs3842787 (n = 3) [4]–[6], *PTGS2* rs5275 (n = 8) [5], [7]–[13], *PTGS2* rs20417 (n = 7) [4], [8]–[10], [12], [14], [15], *PTGS2* rs689466 (n = 3) [8], [11], [12], and rs2745557 (n = 3) [5], [9], [16].

**Table 1:** Summary of articles included in the meta-analysis.

Study	Country	Outcome	Study design		Age		Gender		case				control			
Males/Females									No		Yes		No		Yes	
*PTGS1* rs3842787									CT+TT/CC		CT+TT/CC		CT+TT/CC		CT+TT/CC	
Hubner *et al*, 2007 [4]	UK	CRA	cohort study		57.3 ± 9.3		289/256		8/66		8/55		20/186		30/173	
Gallicchio *et al*, 2006 [5]	USA	BC	cohort study		53.2		0/1467 (females only)		10/55		2/13		136/770		51/305	
Ulrich *et al*, 2004 [6]	USA	CRA	case-control study		30-74		Without details		41/287		29/190		56/288		38/273	
*PTGS2* rs5275									TC+CC/ TT		TC+CC/ TT		TC+CC/ TT		TC+CC/ TT	
Lurie *et al*, 2010 [7]	USA	OC	case-control study		≥18		0/2454 (females only)		300/282		194/172		452/375		344/361	
Andersen *et al*, 2009 [8]	Denmark	CRC	cohort study		50-64		619/505		151/94		61/53		306/222		144/93	
Barry *et al*, 2009 [9]	USA	CRA	cohort study		57.6 ± 9.6		630/349		81/72		156/118		103/70		200/163	
Gong *et al*, 2009 [10]	USA	CRA	case-control study		30-74		168/205		84/50		14/14		96/54		46/15	
Vogel *et al*, 2008 [11]	Denmark	LC	nested case-cohort study		50-64		631/516		151/125		69/54		290/218		139/90	
Vogel *et al*, 2007 [12]	Denmark	BCC	nested case-cohort study		50-64		293/326		131/92		49/29		120/97		49/46	
Vogel *et al*, 2006 [13]	Denmark	BC	nested case-cohort study		50-64		0/712 (females only)		83/73		108/92		84/50		119/103	
Gallicchio *et al*, 2006 [5]	USA	BC	cohort study		53.2		0/1467 (females only)		37/29		5/9		511/396		198/158	
***PTGS2*** ** rs20417**									**GC+CC/GG**		**GC+CC/GG**		**GC+CC/GG**		**GC+CC/GG**	
Daraei *et al*, 2012 [14]	Iran	CRC	case-control study	58.2±14.8		117/113		64/31		8/7		47/44		19/10		
Andersen *et al*, 2009 [8]	Denmark	CRC	cohort study	50–64		619/505		65/180		27/87		131/397		68/169		
Barry *et al*, 2009 [9]	USA	CRA	cohort study	57.6±9.6		630/349		40/109		86/181		47/117		97/263		
Gong *et al*, 2009 [10]	USA	CRA	case-control study	30–74		168/205		45/89		9/19		60/90		24/37		
Hubner *et al*, 2007 [4]	UK	CRA	cohort study	57.3±9.3		289/256		19/55		19/44		49/157		49/154		
Vogel *et al*, 2007 [12]	Denmark	BCC	nested case-cohort study	50–64		293/326		59/164		25/53		49/168		23/72		
Ulrich *et al*, 2005 [15]	USA	CRA	case-control study	30–74		Without details		95/217		64/127		96/228		83/177		
***PTGS2*** ** rs689466**									**AG+GG/AA**		**AG+GG/AA**		**AG+GG/AA**		**AG+GG/AA**	
Andersen *et al*, 2009 [8]	Denmark	CRC	cohort study	50–64		619/505		89/156		40/74		199/329		84/153		
Vogel *et al*, 2008 [11]	Denmark	LC	nested case-cohort study	50–64		631/516		90/186		49/74		194/314		81/148		
Vogel *et al*, 2007 [12]	Denmark	BCC	nested case-cohort study	50–64		293/326		79/144		25/53		91/126		42/53		
***PTGS2*** ** rs2745557**									**GA+AA/GG**		**GA+AA/GG**		**GA+AA/GG**		**GA+AA/GG**	
Barry *et al*, 2009 [9]	USA	CRA	cohort study	57.6±9.6		630/349		50/105		89/187		59/113		114/255		
Cheng *et al*, 2007 [16]	USA	PC	case-control study	Without details		1337/0 (males only)		64/264		78/413		80/144		108/186		
Gallicchio *et al*, 2006 [5]	USA	BC	cohort study	53.2		0/1467 (females only)		19/50		8/10		306/631		123/239		

Abbreviations: No, non-NSAID users; Yes, NSAID users; PC, prostate cancer; CRC, colorectal cancer; OC, ovarian cancer; CRA, colorectal adenoma; LC, lung cancer; BCC, basal cell carcinoma; BC, breast cancer.

The Hardy-Weinberg equilibrium could not be estimated because the allele frequencies were not clarified in the literature.

### Meta-analysis of the *PTGS1* Polymorphisms and NSAID Use on the Risk of Developing Cancer

**Figure 2:**
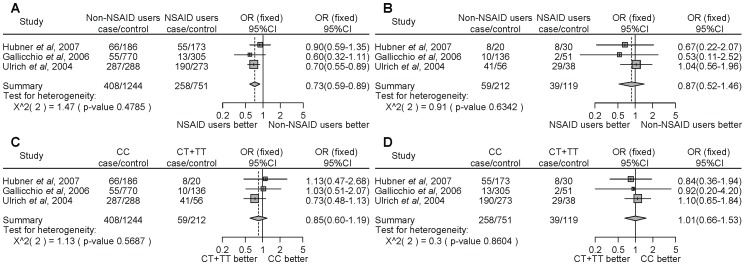
Forest plot of the association between the *PTGS1* rs3842787 polymorphism and NSAID use on cancer risk. The difference in the development of cancer between NSAID use and non-NSAID use from individuals homozygous for the major allele (a), between NSAID use and non-NSAID use from individuals with minor allele carriers (b), between the non-NSAID users homozygous for the major allele and the minor allele carriers (c), and between the NSAID users homozygous for the major allele and the minor allele carriers (d). Squares represent study-specific ORs; horizontal lines represent 95% CIs; size of square reflects study-specific statistical weight (inverse of the variance); diamonds represent summary OR and 95% CI.

For *PTGS1* rs3842787, NSAID users homozygous for the major allele (CC) demonstrated a significantly decreased cancer risk compared with non-NSAID users (Fig. 2A, OR = 0.73, 95% CI = 0.59–0.89). However, there were no significant differences in the risk of developing cancer between NSAID users and non-NSAID users with minor allele carriers (CT+TT) (Fig. 2B, OR = 0.87, 95% CI = 0.52–1.46). There was no significant difference between homozygous for the major allele or carriers of the minor allele among non-NSAID (Fig. 2C, OR = 0.85, 95% CI = 0.60–1.19) or NSAID (Fig. 2D, OR = 1.01, 95% CI = 0.66–1.53) users. We did not detect any significant heterogeneity.

### Meta-analysis of the *PTGS2* Polymorphisms and NSAID Use on the Risk of Developing Cancer

**Figure 3:**
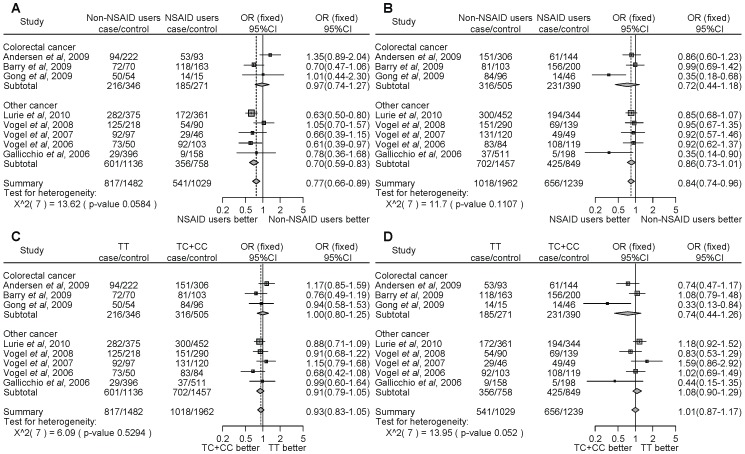
Forest plot of the association between the *PTGS2* rs5275 polymorphism and NSAID use on cancer risk stratified by the type of cancer and overall incidence of cancer. The difference in the development of cancer between NSAID users and non-NSAID users homozygous for the major allele (a), between NSAID users and non-NSAID users with minor allele carriers (b), between the non-NSAID users homozygous for the major allele and the minor allele carriers (c), and between the NSAID users homozygous for the major allele and the minor allele carriers (d). Squares represent study-specific ORs; horizontal lines represent 95% CIs; size of square reflects study-specific statistical weight (inverse of the variance); diamonds represent summary OR and 95% CI.

**Figure 4:**
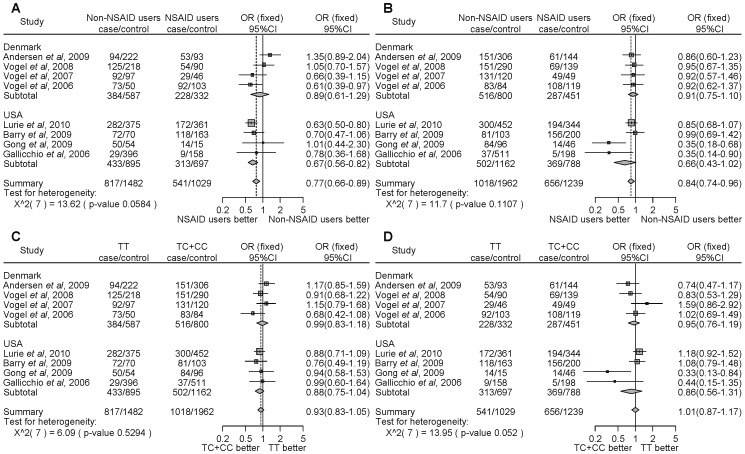
Forest plot of the association between the *PTGS2* rs5275 polymorphism and NSAID use on cancer risk stratified by ethnicity. The difference in the development of cancer between NSAID users and non-NSAID users homozygous for the major allele (a), between NSAID users and non-NSAID users with minor allele carriers (b), between the non-NSAID users homozygous for the major allele and the minor allele carriers (c), and between the NSAID users homozygous for the major allele and the minor allele carriers (d). Squares represent study-specific ORs; horizontal lines represent 95% CIs; size of square reflects study-specific statistical weight (inverse of the variance); diamonds represent summary OR and 95% CI.

For *PTGS2* rs5275, NSAID users significantly decreased the cancer risk compared with non-NSAID users homozygous for the major allele (TT) (Fig. 3A, OR = 0.77, 95% CI = 0.66–0.89). Similarly, NSAID users significantly decreased the cancer risk compared with non-NSAID users with the minor allele carriers (TC+CC) (Fig. 3B, OR = 0.84, 95% CI = 0.74–0.96). However, there were no associations with the *PTGS2* rs5275 polymorphism and NSAID use on the risk of developing cancer (Fig. 3C, D). Thus, the results of the meta-analysis among the 8 studies indicate that NSAID use significantly decreased cancer risk compared with non-NSAID use, despite the *PTGS2* polymorphism. In the stratified analysis by the type of cancer, there were no associations with colon cancer (Fig. 3A–D). However, NSAID users, in contrast to non-NSAID users, homozygous for the major allele, demonstrated a statistically significant decrease of cancers other than colon cancer (Fig. 3A, OR = 0.70, 95% CI = 0.59–0.83). In the subgroup analysis by locality, there were no associations among people of Denmark (Fig. 4A–D). In the USA, NSAID users, in contrast to non-NSAID users, homozygous for the major allele, demonstrated a statistically significant decrease of cancer. (Fig. 4A, OR = 0.67, 95% CI = 0.56–0.82). We did not detect any significant heterogeneity.

**Figure 5:**
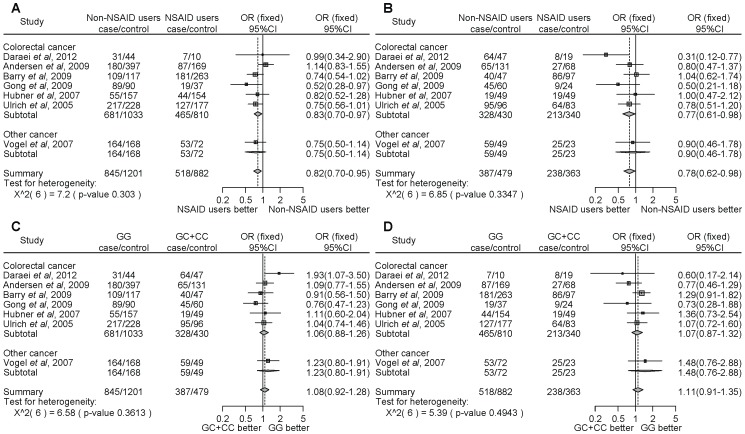
Forest plot of the association between the *PTGS2* rs20417 polymorphism and NSAID use on cancer risk stratified by the type of cancer and overall incidence of cancer. The difference in the development of cancer between NSAID users and non-NSAID users homozygous for the major allele (a), between NSAID users and non-NSAID users with minor allele carriers (b), between the non-NSAID users homozygous for the major allele and the minor allele carriers (c), and between the NSAID users homozygous for the major allele and the minor allele carriers (d). Squares represent study-specific ORs; horizontal lines represent 95% CIs; size of square reflects study-specific statistical weight (inverse of the variance); diamonds represent summary OR and 95% CI.

**Figure 6:**
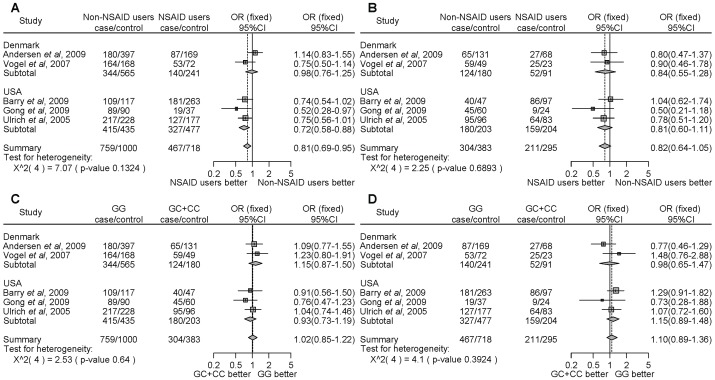
Forest plot of the association between the *PTGS2* rs20417 polymorphism and NSAID use on cancer risk stratified by ethnicity. The difference in the development of cancer between NSAID users and non-NSAID users homozygous for the major allele (a), between NSAID users and non-NSAID users with minor allele carriers (b), between the non-NSAID users homozygous for the major allele and the minor allele carriers (c), and between the NSAID users homozygous for the major allele and the minor allele carriers (d). Squares represent study-specific ORs; horizontal lines represent 95% CIs; size of square reflects study-specific statistical weight (inverse of the variance); diamonds represent summary OR and 95% CI.

For *PTGS2* rs20417, NSAID use significantly decreased cancer risk compared with non-NSAID use in individuals homozygous for the major allele (GG) (Fig. 5A, OR = 0.82, 95% CI = 0.70–0.95). Similarly, NSAID use significantly decreased cancer risk compared with non-NSAID use in individuals with the minor allele carriers (GC+CC) (Fig. 5B, OR = 0.78, 95% CI = 0.62–0.98). However, there were no associations with the risk of developing cancer with NSAID use and the *PTGS2* rs20417 polymorphism (Fig. 5C, D). Thus, the results of the meta-analysis among the 7 studies also indicate that NSAID use significantly decreased cancer risk compared with non-NSAID use, regardless of the *PTGS2* polymorphism. In the stratified analysis by the type of cancer, NSAID users, in contrast to non-NSAID users, homozygous for the major allele or carriers of the minor allele, demonstrated a statistically significantly decrease in colon cancer risk (Fig. 5A, OR = 0.83, 95% CI = 0.70–0.97; Fig. 5B, OR = 0.77, 95% CI = 0.61–0.98, respectively). In the subgroup analysis by locality, there were no associations among people from Denmark (Fig. 6A–D). In the USA, NSAID users, in contrast to non-NSAID users, homozygous for the major allele demonstrated a statistically significant decrease of cancer (Fig. 6A, OR = 0.72, 95% CI = 0.58–0.88).

**Figure 7:**
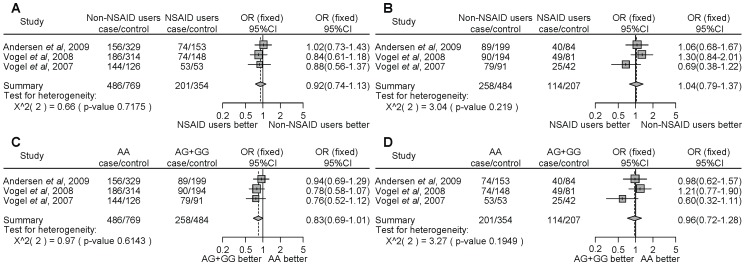
Forest plot of the association between the *PTGS2* rs689466 polymorphism and NSAID use on cancer risk. The difference in the development of cancer between NSAID users and non-NSAID users homozygous for the major allele (a), between NSAID users and non-NSAID users with minor allele carriers (b), between the non-NSAID users homozygous for the major allele and the minor allele carriers (c), and between the NSAID users homozygous for the major allele and the minor allele carriers (d). Squares represent study-specific ORs; horizontal lines represent 95% CIs; size of square reflects study-specific statistical weight (inverse of the variance); diamonds represent summary OR and 95% CI.

**Figure 8:**
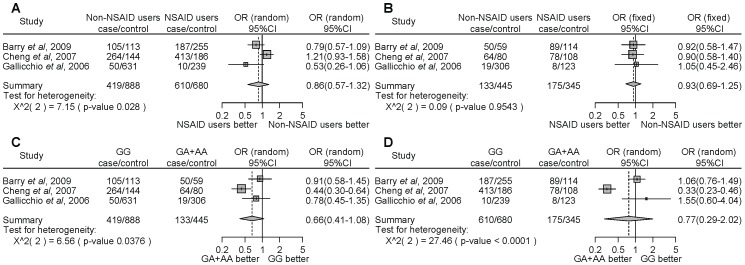
Forest plot of the association between the *PTGS2* rs2745557 polymorphism and NSAID use on cancer risk. The difference in the development of cancer between NSAID users and non-NSAID users homozygous for the major allele (a), between NSAID users and non-NSAID users with minor allele carriers (b), between the non-NSAID users homozygous for the major allele and the minor allele carriers (c), and between the NSAID users homozygous for the major allele and the minor allele carriers (d). Squares represent study-specific ORs; horizontal lines represent 95% CIs; size of square reflects study-specific statistical weight (inverse of the variance); diamonds represent summary OR and 95% CI.

For *PTGS2* rs689466 and rs2745557, we found that there were no associations between the risk of developing cancer and NSAID use and polymorphisms (Fig. 7A–D and Fig. 8A–D).

### Sensitivity Analyses

For *PTGS1* rs3842787, sensitivity analyses indicated that the results of one independent study by Ulrich *et al*. [6] affected our original results considerably, and inclusion of this study was primarily responsible for the significant difference observed in the risk of cancer development between NSAID users and non-NSAID users homozygous for the major allele. For *PTGS2* rs5275, sensitivity analyses indicated that inclusion of the independent study by Lurie *et al*. [7] was primarily responsible for the significant difference observed in the risk of cancer development between NSAID users and non-NSAID users homozygous for the major allele in the overall group, cancer subgroups other than colon cancer, and the USA subgroup. Similarly, inclusion of the independent study by Barry *et al*. [9] was mainly responsible for our original results in which no associations were observed between gene polymorphism and the risk of cancer development among NSAID users in the colon cancer subgroup. For *PTGS2* rs20417, sensitivity analyses indicated that inclusion of the independent studies by Barry *et al*. [9], Gong *et al*. [10], and Ulrich *et al*. [15] was responsible for the significant difference observed in the risk of cancer development between NSAID users and non-NSAID users homozygous for the major allele in the colon cancer subgroup. In addition, inclusion of independent studies by Daraei *et al*. [14], Gong *et al*. [10], and Ulrich *et al*. [15] was found to be primarily responsible for the significant difference in the risk of cancer development between NSAID users and non-NSAID users with minor allele carriers in the overall group and the colon cancer subgroup. For *PTGS2* rs689466, sensitivity analyses indicated that inclusion of the independent study by Andersen *et al*. [8] was mainly responsible for our original results in which no associations were observed between gene polymorphism and the risk of cancer development among non-NSAID users. For *PTGS2* rs2745557, sensitivity analyses indicated that the results of one independent study by Cheng *et al*. [16] were primarily responsible for no significant difference being observed in the risk of cancer development between NSAID users and non-NSAID users homozygous for the major allele. These results suggest that a limited number of studies could substantially influence the ORs.

### Publication Bias

Begg’s test and Egger’s test were performed to estimate the publication bias of the literature (Table 2). Egger’s test did not indicate any evidence of potential publication bias; Begg’s test indicated that publication biases generally have no significant effect on the results of overall analysis, except for the association between the *PTGS2* rs5275 polymorphism and NSAID users (P = 0.026), which was most likely due to the limited number of studies on *PTGS2* rs5275 polymorphism.

**Table 2:** Egger’s and Begg’s test to measure the funnel plot asymmetric.

Polymorphisms				
*PTGS1* rs3842787	No vs. Yes (CC)	No vs. Yes (CT+TT)	CC vs. CT+TT (No)	CC vs. CT+TT (Yes)
P_E_	0.987	0.075	0.101	0.527
P_B_	0.602	0.117	0.117	0.602
*PTGS2* rs5275	No vs. Yes (TT)	No vs. Yes (TC+CC)	TT vs. TC+CC (No)	TT vs. TC+CC (Yes)
P_E_	0.415	0.071	0.844	0.066
P_B_	0.458	0.322	1.000	**0.026**
*PTGS2* rs20417	No vs. Yes (GG)	No vs. Yes (GC+CC)	GG vs. GC+CC (No)	GG vs. GC+CC (Yes)
P_E_	0.622	0.183	0.604	0.313
P_B_	0.881	0.293	0.652	0.293
*PTGS2* rs689466	No vs. Yes (AA)	No vs. Yes (AG+GG)	AA vs. AG+GG (No)	AA vs. AG+GG (Yes)
P_E_	0.847	0.150	0.680	0.155
P_B_	0.602	0.117	0.602	0.117
*PTGS2* rs2745557	No vs. Yes (GG)	No vs. Yes (GA+AA)	GG vs. GA+AA (No)	GG vs. GA+AA (Yes)
P_E_	0.379	0.065	0.431	0.768
P_B_	0.117	0.117	0.602	0.602

Abbreviations: No, non-NSAID users; Yes, NSAID users; P_E_: P for Egger’s test, P_B_; P for Begg’s test.

The bold value indicates a potential publication bias.

## Discussion

In the current study, we searched the literature to determine the association between *PTGS1* or *PTGS2* polymorphisms and NSAID use on the risk of developing cancer. Although many SNPs located in the region of *PTGS1* are known, 1 polymorphism (rs3842787) was analyzed by 3 independent researchers to determine whether the gene polymorphism and NSAID use is associated with cancer risk. Ulrich *et al*. [6] reported that NSAID use by individuals with the wild type polymorphism of *PTGS1* rs3842787 had a significantly reduced (Fig. 2A, OR = 0.70, 95% CI = 0.55–0.89) adenoma risk compared with non-NSAID users. However, Gallicchio *et al*. [5] and Hubner *et al*. [4] reported that there was no association between the *PTGS1* rs3842787 polymorphism and NSAID use on the development of cancer. Our meta-analysis showed that the NSAID users had a lower risk of developing cancer compared with the non-NSAID users among individuals homozygous for the major allele of *PTGS1* rs3842787. The rs3842787 SNP is located in exon 2 of *PTGS1*, and causes the substitution of a leucine for a proline at codon 17 (P17L). These results suggest that the *PTGS1* rs3842787 non-synonymous polymorphism may be an important pharmacogenomic biomarker.

For *PTGS2*, there have been studies of 4 SNPs (rs5275, rs20417, rs689466, and rs2745557), which were analyzed for an association with cancer risk and NSAID use; however, the studies have produced mixed results. The rs5275 SNP is located in exon 10 (3′-untranslated region: 3′-UTR) of the *PTGS2* gene, which is downstream of the stop codon, and the C allele has been associated with lower steady-state *PTGS2* mRNA levels [7]. The rs20417 SNP is located in the promoter region of the *PTGS2* gene. The C variant allele of the rs20417 has significantly lower promoter activity than the G allele [10]. In a recent meta-analysis study, the rs20417 emerged to be an influential SNP on colorectal cancer risk in the Asian population [17]. The rs689466 SNP is also located in the promoter region of the *PTGS2* gene. The A allele of the rs689466 has been associated with strikingly higher promoter activity [18]. Dong *et al*. [19] reported that the A allele of rs689466 was significantly associated with increased risk of digestive system cancers. The location of these polymorphisms on the gene promoter region would directly influence the regulation of gene expression and the rate of enzyme production [14]. Therefore, it is considered that these polymorphisms, in conjunction with NSAID use, have an influence on cancer risk; however, our meta-analysis did not detect associations in any group. On the other hand, we found that the associations between *PTGS2* polymorphisms and NSAID use on cancer risk differ by the type of cancer and ethnicity. Because *PTGS2* is not constitutively expressed in tissues but is induced by growth factors, inflammatory cytokines, and tumor promoters, the effect of NSAIDs on *PTGS2* may differ by tissues. Furthermore, Zhang *et al*. [20] found that the haplotype of *PTGS2* including rs20417 and rs689466 SNP was associated with gastric cancer in Chinese populations, which indicates the necessity to study haplotypes.

In these studies, the types of NSAIDs (e.g., aspirin, ibuprofen, and other NSAIDs), dose methods (e.g., dosage and duration), study design (e.g., case control study or cohort study), population (e.g., age, gender, type of cancer, and ethnic), and study power are different. In addition, there was the lack of specificity for cancer type in our analysis because few studies have investigated the effect of associations between polymorphisms in *PTGS1* and *PTGS2* genes and NSAID use on cancer risk. Thus, it is difficult to draw any conclusion about the relationship between *PTGS* genotype and NSAID use on the risk of developing cancer. Nonetheless, our results provide limited evidence. Drug response is a complex phenomenon dependent on inherited and environmental factors. To carry more credibility, further analyses with study design formulation are required in several countries.

## Supporting Information

Table S1 Characteristics of studies included in the meta-analysis.
(XLSX)
